# Development of a prognostic model based on an immunogenomic landscape analysis of medulloblastoma

**DOI:** 10.1042/BSR20202907

**Published:** 2021-01-07

**Authors:** Yuduo Guo, Shenglun Li, Peng Huang, Hongwei Zhang, Chunjiang Yu

**Affiliations:** Department of Neurosurgery, Sanbo Brain Hospital, Capital Medical University, No.50 Yikesong Road, Haidian District, 100093 Beijing, P.R. China

**Keywords:** a prognostic model, immune-related genes, integrated analysis, medulloblastoma

## Abstract

Medulloblastoma (MB) is one of the most common central nervous system tumors in children. At present, the vital role of immune abnormalities has been proved in tumorigenesis and progression. However, the immune mechanism in MB is still poorly understood. In the present study, 51 differentially expressed immune-related genes (DE-IRGs) and 226 survival associated immune-related genes (Sur-IRGs) were screened by an integrated analysis of multi-array. Moreover, the potential pathways were enriched by functional analysis, such as ‘cytokine–cytokine receptor interaction’, ‘Ras signaling pathway’, ‘PI3K-Akt signaling pathway’ and ‘pathways in cancer’. Furthermore, 10 core IRGs were identified from DE-IRGs and Sur-IRGs. And the potential regulatory mechanisms of core IRGs were also explored. Additionally, a new prognostic model, including 7 genes (HDGF, CSK, PNOC, S100A13, RORB, FPR1, and ICAM2) based on IRGs, was established by multivariable COX analysis. In summary, our study revealed the underlying immune mechanism of MB. Moreover, we developed a prognostic model associated with clinical characteristics and could reflect the infiltration of immune cells.

## Introduction

Medulloblastoma accounts for 20% of pediatric central nervous system tumors. The standard treatment for MB is surgical resection, assisted by radiotherapy and chemotherapy [1,2]. Despite aggressive treatment, approximately 50% of MBs metastasize in the central nervous system in early-stage [3]. Additionally, the side effects of radiotherapy and chemotherapy are inevitable [4].

Over the past decade, emerging studies have demonstrated that tumor immunity plays a crucial role in the malignant progression of tumors [5–7]. Moreover, as an effective treatment by leveraging the immune system, immunotherapy has shown excellent antitumor effects in a variety of tumors [8,9]. For instance, Nivolumab and Pembrolizumab targeting programmed death-1 (PD-1) have effectively treated melanoma, lung cancer, and Hodgkin lymphoma [10–13]. As well as Ipilimumab, as an inhibitor of cytotoxic T lymphocyte-associated antigen-4 (CTLA-4), has been proved to be valid in treating melanoma and lung cancer [14,15]. However, the immunotherapy application for MB is still limited, attributed to the poorly understood of MB’s immune mechanisms. Moreover, the immunophenotypes were various in different types of tumors [16]. Therefore, it is necessary to study MB’s underlying immune mechanisms, including immune-related genes (IRGs) and immune cell infiltration.

For the first time in the present study, we screened the differentially expressed immune-related genes (DE-IRGs) and survival associated immune-related genes (Sur-IRGs). Furthermore, by multiple computational methods, we developed and validated a prognostic model based on IRGs, which could adequately reflect MB's prognosis and immune cell infiltration. In summary, our results were expected to promote immunotherapy and the personalized treatment of MB.

## Results

### Screening and functional analysis of DE-IRGs in medulloblastoma

**Figure 1:**
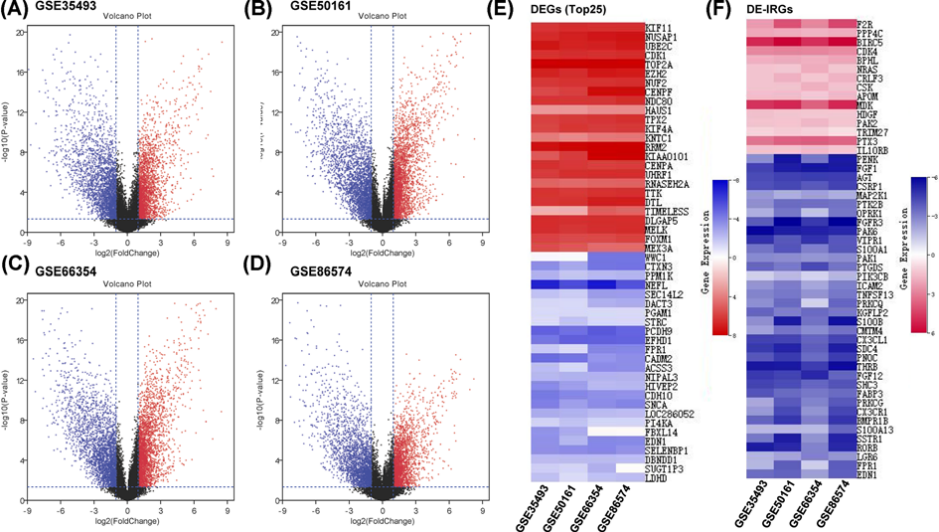
Screening of DE-IRGs by integrated analysis (**A**–**D**) Volcano plots of DEGs between MB and normal tissues in training data sets, *P*-value < 0.05, |log2 (Fold Change)| > 1. (**E**) Heatmap of top 25 DEGs integrated by RRA algorithm from multiple data sets. (**F**) Heatmap of DE-IRGs of training data sets.

To clarify the immunogenomics characteristics in MB, as shown in Figure 1A–D (Supplementary Figure S1 and Supplementary Files 1–4), thousands of differentially expressed genes (DEGs) were screened from four independent data sets by comparing MB with normal tissues. Then, 1510 DEGs, including 780 up- and 730 down-regulated, were obtained by the RRA algorithm (Figure 1E). Moreover, 51 differentially expressed immune-related genes (DE-IRGs) were selected from DEGs, including 15 up- and 36 down-regulated (Figure 1F and Supplementary Figure S2). These results indicated that the expression profile of MB is significantly irregular, including IRGs.

**Figure 2:**
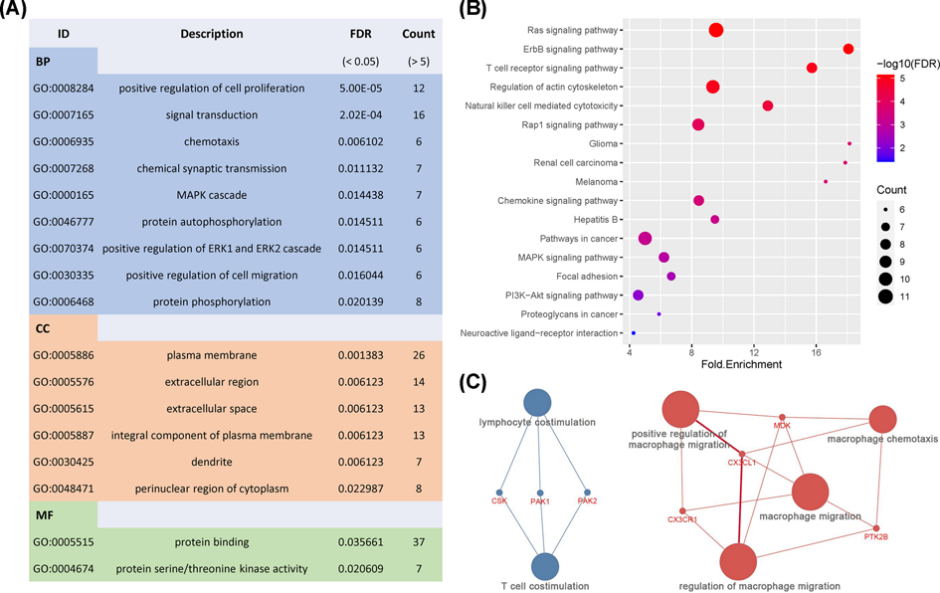
Functional analysis of DE-IRGs (**A**) Gene ontology analysis of DE-IRGs, BP for biological process, CC for cellular component, and MF for molecular function. (**B**) Enriched KEGG pathways of DE-IRGs (FDR < 0.05 and count >5). (**C**) Immune System Process enrichment of DE-IRGs.

To further define the function of DE-IRGs in MB, functional analysis was performed to reveal the underlying mechanism. As shown in Figure 2A, for BP term, DE-IRGs were mainly enriched in tumor-related processes, such as positive regulation of ERK1/ ERK2 cascade, JAK-STAT cascade, angiogenesis, cell proliferation, and migration. For CC term, DE-IRGs were primarily enriched in the extracellular region and cell–cell junction. These genes were mainly involved in ATP binding, protein serine/threonine kinase activity, and protein kinase C activity for the MF term.

Besides, as shown in Figure 2B, the DE-IRGs were mainly involved in tumor-related pathways, including Ras, PI3K-Akt, and MAPK signaling pathways. Importantly, immune-related pathways were also involved, such as NK cell-mediated cytotoxicity, chemokine, and T-cell receptor signaling pathways. Moreover, the Immune System Process enrichment indicated the possibility of dysregulation of lymphocytes and macrophages in MB’s tumor microenvironment (Figure 2C).

### Identification and analysis of Sur-IRGs

**Figure 3:**
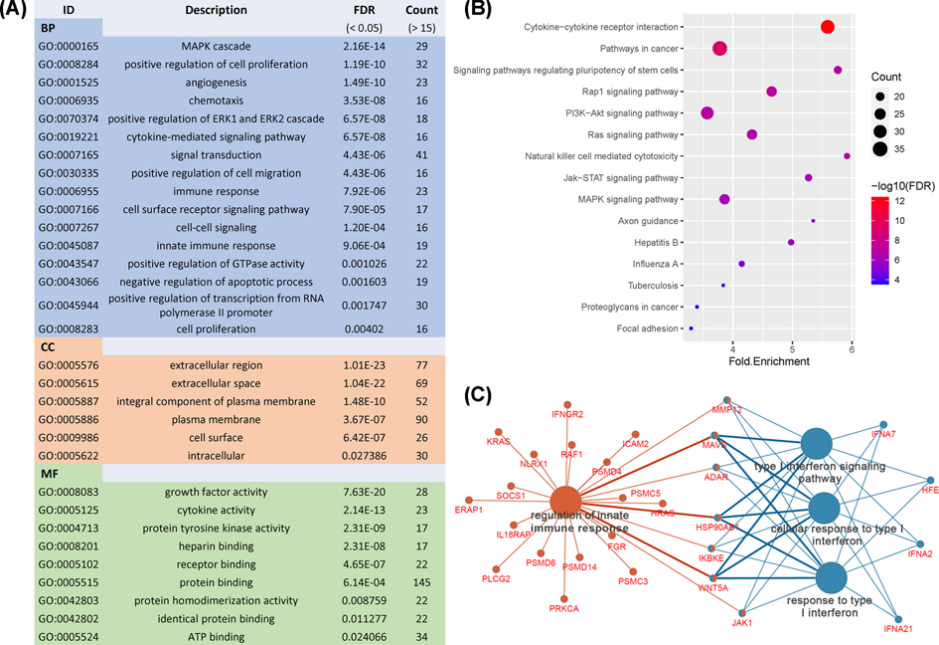
Functional analysis of Sur-IRGs (**A**) Gene ontology analysis of Sur-IRGs. (**B**) Enriched KEGG pathways of Sur-IRGs (FDR < 0.05 and count > 15). (**C**) Immune System Process enrichment of Sur-IRGs.

In addition, 226 Sur-IRGs were identified by survival analysis from another independent data set containing 605 MBs. Then, functional analysis was performed to uncover the underlying mechanisms of Sur-IRGs. Similar to results from the analysis of DE-IRGs, as shown in Figure 3A, Sur-IRGs were primarily enriched in several GO terms related to tumor progression and immune regulation, including cytokine-mediated signaling pathway, immune response, and cytokine activity.

Significantly, for the KEGG pathway (Figure 3B), Sur-IRGs were mainly involved in PI3K-Akt, Ras, MAPK, Jak-STAT signaling pathways, cytokine–cytokine receptor interaction, and natural killer cell-mediated cytotoxicity, which play vital roles in tumor progression and immune regulation. Besides, we found that innate immune response and type I interferon may have an important impact on MB patients’ prognosis (Figure 3C).

### Identification and characteristics of core IRGs

**Figure 4:**
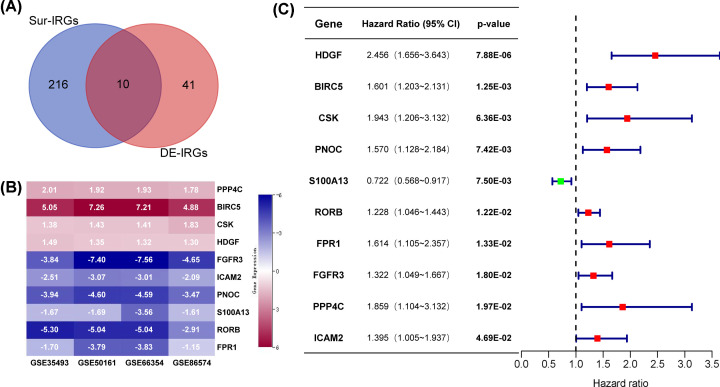
Identification of core IRGs (**A**) Venn diagram of Sur-IRGs and DE-IRGs. (**B**) Expression of core IRGs in training data sets. (**C**) Forest plot of hazard ratios showing the prognostic values of core IRGs.

To identify IRGs which were differentially expressed in MB and related to prognosis, 10 core IRGs were selected from DE-IRGs and Sur-IRGs (Figure 4A). As shown in Figure 4B, 4 core IRGs, including PPP4C, BIRC5, CSK, and HDGF, were up-regulated, and 6 core IRGs, including FGFR3, ICAM2, PNOC, S100A13, RORB, and FPR1, were down-regulated in MB. A forest plot of hazard ratios indicated that most of the core IRGs were tumor promoters (Figure 4C and Supplementary Figure S4).

### Construction of TF regulatory network

**Figure 5:**
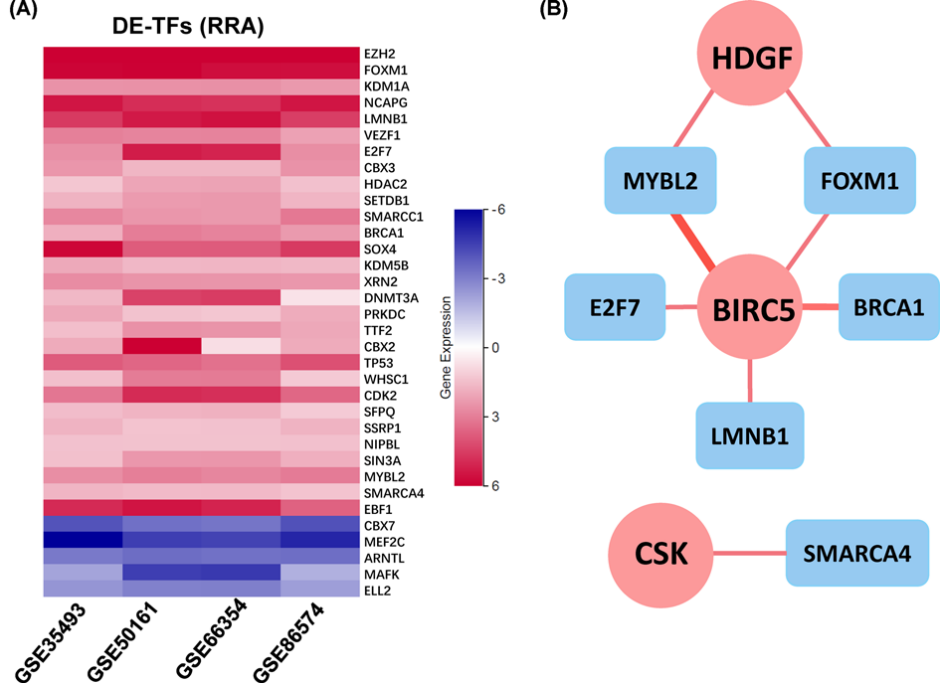
Construction of TF regulatory network (**A**) Differentially expressed TFs (*P*-value < 0.05 and |log(fold change)| >1). (**B**) Regulatory network constructed based on DE-TFs (blue) and core IRGs (red).

To explore potential regulatory mechanisms of core IRGs, 34 out of 318 transcription factors (TFs) were found differentially expressed between MB and normal samples. As shown in Figure 5A, most of the DE-TFs were overexpressed in MB. Then, a regulatory network shown in Figure 5B was constructed based on relationships between DE-TFs and core IRGs. These results indicated that core IRGs could be affected by TFs to regulate immune functions.

### Construction of a clinical prognostic model

To illustrate the prognostic value of IRGs, seven IRGs out of core IRGs were selected to construct a prognostic model by multivariate Cox regression analysis (Supplementary Table S1). The formula of the prognostic model was as follows:

$$\begin{array}{l} \text{Risk}\;\text{score}=[\text{HDGF}\times 1.128]+[\text{CSK}\times 0.489]+[\text{PNOC}\times 0.389]+[\text{RORB}\times 0.243]+[\text{FPR}1\times 0.313]+\\ \left[\text{ICAM}2\times 0.376]+[\text{S}100\text{A}13\times (-0.379)\right] \end{array}$$

**Figure 6:**
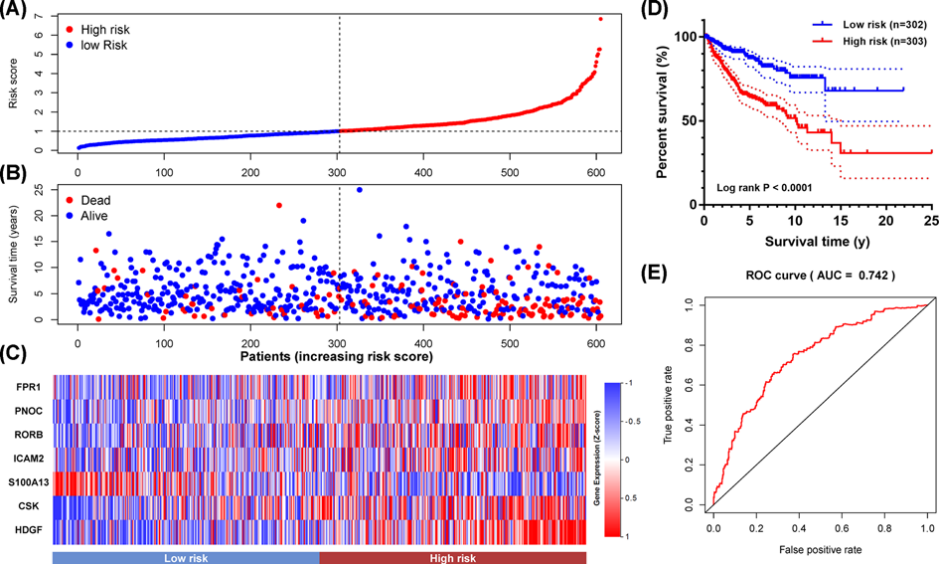
The prognostic value of the prognostic model (**A**) The rank of the risk score and distribution of groups. (**B**) Survival status of MBs with different risk scores. (**C**) Heatmap of expression profiles of genes in the prognostic model. (**D**) Survival analysis of MBs with different prognostic signatures. (**E**) Survival-dependent ROC curve of the prognostic signature.

As shown in Figure 6A–C, MBs were divided into two groups with high- and low-risk scores. The survival analysis showed that the prognostic model could distinguish among MB patients based on potential discrete outcomes (Figure 6D). Moreover, the area under the curve (AUR) of the ROC curve was 0.742, indicating a moderate capability for the prognostic model in survival monitoring (Figure 6E).

### Validation of the IRG-based prognostic model

**Figure 7:**
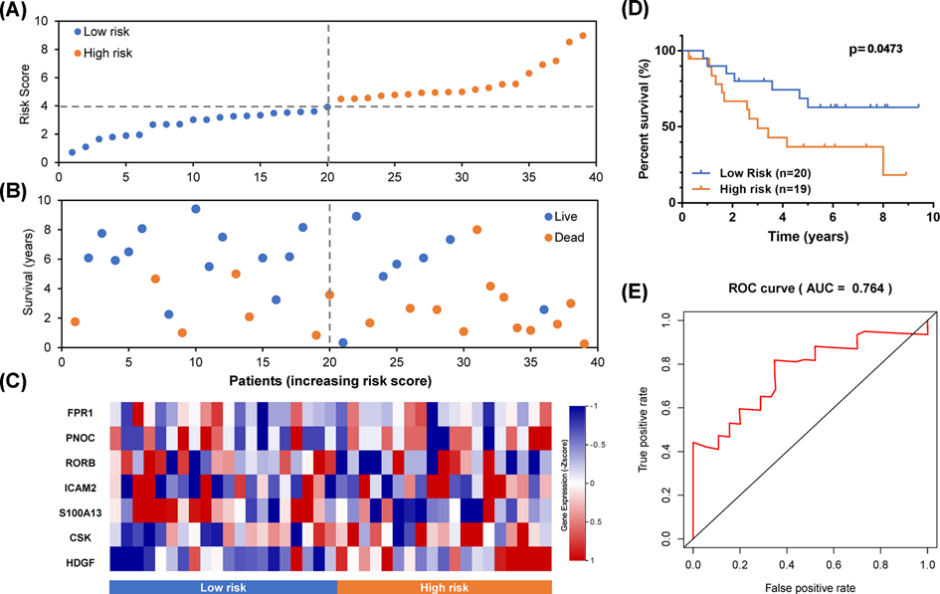
Validation of the prognostic model in a test set (**A**) The rank of the risk score and distribution of groups. (**B**) Survival status of MBs with different risk scores. (**C**) Heatmap of expression profiles of included genes in the prognostic model. (**D**) Survival analysis of MBs with different prognostic signatures. (**E**) Survival-dependent ROC curve of the prognostic signature.

For excluding false positives of the prognostic model of MBs, another independent data set was introduced as a test set. The risk score of each sample was calculated using the formula of this prognostic model. As shown in Figure 7A–C, samples were divided into two groups according to risk scores. By survival analysis, the prognosis of MBs with different risks was significantly different (Figure 7D). Moreover, in Figure 7E, the AUC of ROC is 0.764, which also proved the prognostic model's accuracy based on IRGs.

### The prognostic model is an independent risk factor for MB

**Figure 8:**
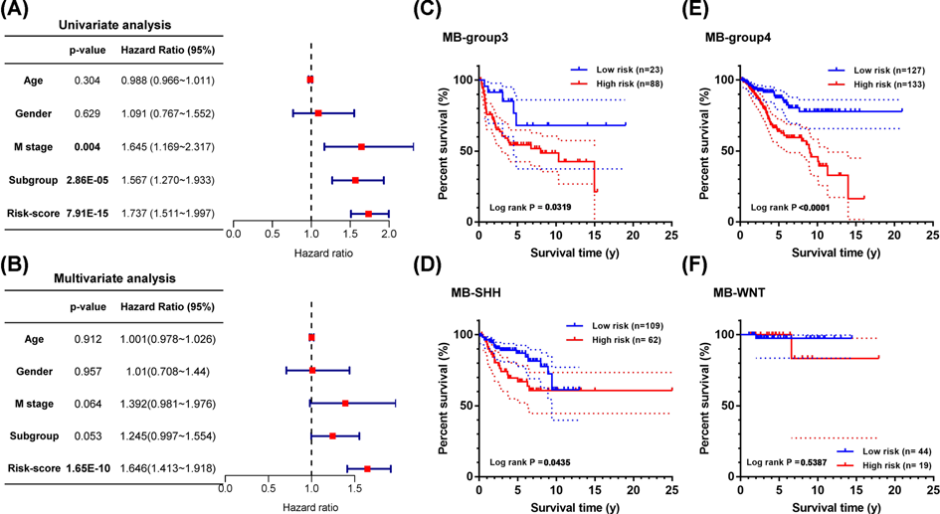
Evaluation of the prognostic value of this model (**A**) Univariate and (**B**) multivariate regression analysis of MB. (**C–F**) Survival analysis of the prognostic model in different subtypes of MB.

Furthermore, univariate and multivariate Cox regression analyses were used to evaluate the prognostic value of the model. As shown in Figure 8A, the metastasis status, subtypes, and the prognostic model (risk score) of MB were significant risk factors by univariate Cox regression analysis. However, in the multivariate Cox regression analysis, the prognostic model (risk score) proved to be the only significant prognostic risk factor (Figure 8B).

Since MB is composed of four subtypes, including WNT, SHH, Group 3, and Group 4, we evaluated the prognostic value of the prognostic model in MBs of different subtypes (Figure 8C–F). Then, by survival analysis, the significant prognostic values were proved in every subtype of MB except for the WNT subtype, which may be due to the small sample size and the favorable prognosis of the WNT subtype (Figure 8F). Besides, the prognostic model also worked well in the classic subtype MB (Supplementary Figure S5).

### Clinical utility of the model and involved core genes

**Figure 9:**
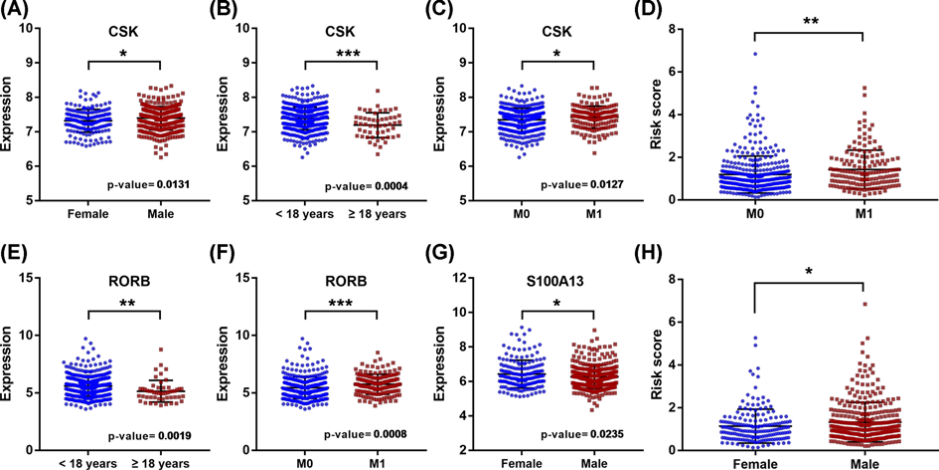
Expression of core genes in MBs with different clinical characteristics (**A–C,E–G**) Relationships between IRGs of the prognostic signature and clinical characteristics in MB; (**D** and** H**) Relationships between the prognostic signature and clinical characteristics in MB (* *P*-value < 0.05; **P-value < 0.01; ***P-value < 0.001)

**Figure 10:**
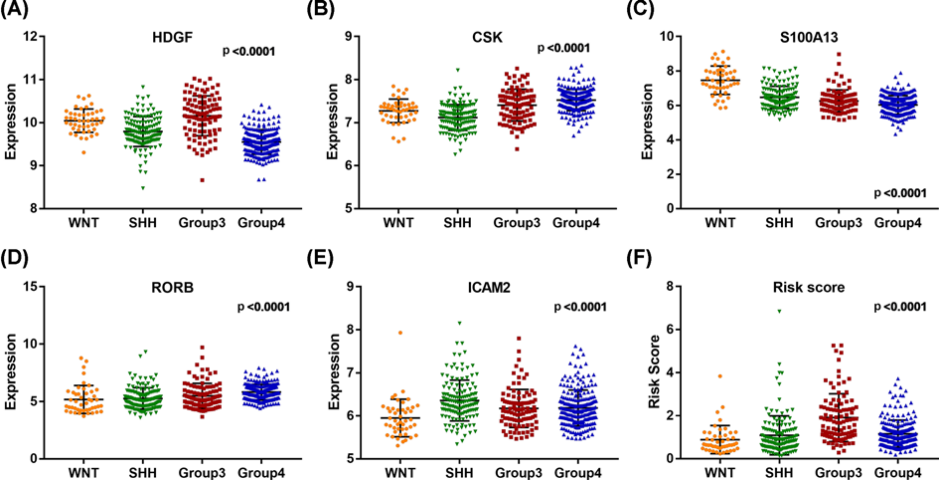
Expression of core genes in different subtypes of MB (**A–E**) Expression levels of IRGs of the prognostic model in different MB subtypes; (**F**) The risk scores of different MB subtypes.

In addition, the relationships between clinical characteristics and the prognostic model, as well as involved IRGs, were analyzed. As shown in Table 1, the expression of CSK was significantly correlated with age, gender, and metastasis stage (Figure 9A–C); the expression of RORB was significantly associated with age and metastasis stage (Figure 9E,F); the expression of S100A13 was significantly correlated with gender (Figure 9G); besides, the risk score was significantly associated with gender and metastasis stage (Figure 9D,H). Additionally, MB’s subtype significantly correlated with risk-score, and almost all IRGs involved in the prognostic signature (Figure 10).

**Table 1:** Relationship between the expressions of the IRGs and the clinicopathological factors in MB

Genes	Age		Gender		M stage		Molecular subgroup
	(≥18 / <18)		(Female/Male)		(M0 / M1)		(Wnt, SHH, group3, grou4)
	*t*	*P*-value	*t*	*P*-value	*t*	*P*-value	*P*-value
**HDGF**	-0.249	0.804	-1.576	0.116	-1.371	0.171	**< 0.0001**
**CSK**	3.774	**3.67E-04**	-2.493	**0.013**	-2.508	**0.013**	**< 0.0001**
**PNOC**	-1.038	0.304	-0.048	0.962	0.245	0.806	0.7866
**S100A13**	-0.072	0.943	2.276	**0.024**	1.183	0.238	**< 0.0001**
**RORB**	3.25	**0.002**	0.744	0.457	-3.398	**7.66E-04**	**< 0.0001**
**FPR1**	0.079	0.938	-0.383	0.702	-0.697	0.486	0.0824
**ICAM2**	-0.535	0.595	0.081	0.935	0.425	0.671	**< 0.0001**
**Risk-Score**	1.016	0.313	-2.429	**0.016**	-2.793	**0.006**	**< 0.0001**

Note: *t* and *P*-values from Student’s *t*-test, M for metastasis.

### Relationship between infiltration immune cells and the prognostic model in MBs

**Figure 11:**
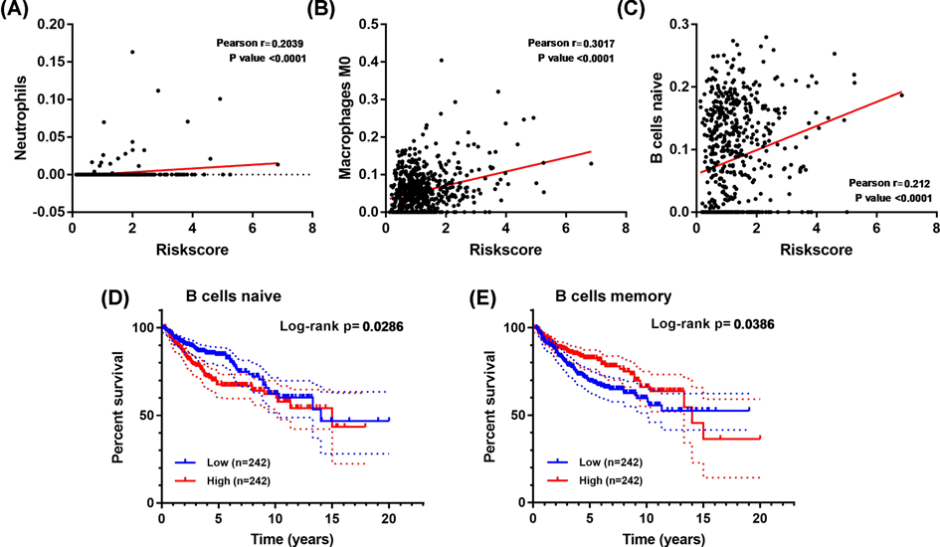
The correlation between risk score and infiltrating immune cells of MB (**A–C**) Relationships between the risk score and infiltration immune cells. (**D** and **E**) Survival analysis of naive B cell and memory B cell levels in MBs.

Due to the prognostic model was constructed based on core IRGs, we estimated the 22 immune infiltrating cells of MBs by the CIBERSORT algorithm. As shown in Figure 11A–C, three types of infiltration immune cells, including neutrophils, macrophages, and naïve B cells, were significantly positively correlated with the risk score. Moreover, survival analysis, naïve B cells, and memory B cells were found to be closely related to the prognosis of MB (Figure 11D,E). These results suggested that the prognostic model reflected the status of infiltrating immune cells, which plays a crucial role in MBs.

## Discussion

In recent years, with the rapid development of tumor immunology, immunotherapy for various tumors has achieved great success. However, research on immunotherapy of MB is still limited, which is attributed to the insufficient understanding of the immune mechanism underlying MB [17]. In the present study, we performed a comprehensive analysis of multiple MB data sets and developed a prognostic model based on IRGs. Our results revealed the underlying immunoregulatory mechanism of MB and interpreted the clinical value of the prognostic model.

First, our results showed that the expression profile of IRGs was significantly different between MB and normal tissues, indicating that MB’s immune mechanism was significantly aberrant. Additionally, numerous IRGs were associated with prognosis, suggesting the critical role of tumor immunity in MB. Then, the functional analysis of IRGs identified several immune-related pathways, including ‘cytokine–cytokine receptor interaction’, ‘NK cell mediated cytotoxicity’, and ‘T-cell reporter signaling pathway’, which suggested the potential immune-related mechanisms underlying MB. Our results also revealed that macrophages’ regulation, innate immune response, and type I interferon might be involved in MBs’ occurrence and development. Cytokines are a type of extracellular soluble protein or glycoprotein released by various stimulated cells and binds to specific surface receptors on target cells, thereby regulating and mobilizing the inflammatory immune response [18]. A previous study revealed that cytokines in MB are different from other brain tumors, including ependymoma, sarcoma, and glioma [19]. Liu’s study indicated that CCL2 secreted by astrocytes contributes to maintaining the stemness of MB [20]. And Chen demonstrated that blocking interleukin-6 (IL-6) signaling inhibits a series of malignant phenotypes of MB cells [21]. Natural killer (NK) cells are equipped with various receptors that could recognize target cells, which triggers NK cell activation and target cell lysis [22]. A recent study demonstrated that NK cells play a significant role in immune defense against tumors, and they are good candidates for new immunotherapeutic approaches [22]. Moreover, Castriconi’s study showed that NK cells are able to lyse MB cell lines [23]. Activation of T lymphocytes is an essential event for an efficient response of the immune system. Under the stimulation of foreign antigens, a series of signal cascades are generated, which induces the proliferation and differentiation of T cell [24]. Patients with MB often present with T-cell lymphopenia [25,26]. Additionally, chimeric antigen receptor (CAR) T-cell therapy has proven effective in multiple studies [27–29]. In addition, innate immunity and type I interferon have also been proved by more and more studies to play an irreplaceable role in tumor progression [30–34]. Combined with these studies, our results indicated that these immune mechanisms might also play an essential role in MB.

In order to clarify the role of IRGs in MB, we constructed a prognostic model. Furthermore, the results of the present study demonstrated the reliable prognostic value of the prognostic model based on IRGs. Moreover, the model and involved genes were associated with a series of characteristics, including age, gender, and metastasis status, which pointed out that tumor immunity differed in different MB patients. Besides, MB classification that has been widely applied in the diagnosis and treatment is based on the genomic characteristics, including WNT, SHH, Group 3, and Group 4 subgroups [35]. Pham and Bockmayr’s studies indicated that the immune microenvironment was significantly different in subtypes of MB [36,37]. Therefore, we investigated the model’s prognostic value and the expression levels of involved IRGs in each MB subgroup. Our results illustrated that scores of the model and the major involved IRGs differed in different MB subgroups, which suggested the difference of tumor immunity in subtypes of MB. Additionally, our results presented a significant prognostic value of the prognostic model in three MB subgroups, except the WNT subgroup, which might be due to the small sample size and the favorable prognosis of this subgroup.

The prognostic model comprises the expression levels of seven IRGs, including FPR1, PNOC, RORB, ICAM2, S100A13, CSK, and HDGF, which have been rarely studied in MB. Formyl peptide receptor (FPR1) is a G protein-coupled receptor (GPCR) mainly expressed in phagocytic leukocytes and is known to play an essential role in host defense and inflammation [38]. Besides, recent studies showed that it is expressed in several types of cancer tissues, such as gastric and colorectal cancer [39,40]. Moreover, FPR1 has been demonstrated to regulate the proliferation, invasion, and angiogenesis of tumor cells [41,42]. Prepronociceptin (PNOC) is a precursor protein of the opioid receptor-like receptor (ORL1) agonist [43]. Moreover, the overexpression expression of PNOC has been identified in other brain tumors [44]. Retinoic acid-related orphan receptor beta (RORB) is a DNA transcription enhancer and has been demonstrated to regulate tumorigenesis by the Wnt-pathway [45]. Moreover, Wen’s study showed that RORB is down-regulated in colorectal cancer [45]. In contrast, our result suggested that RORB was up-regulated in MB, indicating that RORB might be involved in a different MB mechanism. Intercellular adhesion molecule 2 (ICAM2) is a transmembrane glycoprotein of the immunoglobulin superfamily expressed on endothelial cells, platelets, and leukocytes [46]. A previous study has proved that ICAM2 is involved in the transmigration of leukocytes [47]. And the transcellular neutrophil diapedesis across the blood-brain barrier is dependent on endothelial ICAM2 [48]. S100 Calcium Binding Protein A13 (S100A13) is an acidic-Ca^2+^ binding protein of the S100 family, which has been proved to be a powerfully angiogenic biomarker for several tumors [49–51]. Furthermore, Ma’s study reported that S100A13 functions in some immune-related signaling pathways, including cytokine and NF-κB signaling [52]. C-Terminal Src Kinase (CSK), an Src tyrosine kinase, is activated by many stimulators, including epidermal growth factor receptor (EGFR), high glucose, and IL-1 signaling [53,54]. Recently, a study reported that CSK is involved in the process of T-cell activation [55]. Hepatoma-derived growth factor (HDGF) is a vital promoter of many cancers, including liver cancer, stomach cancer, and lung cancer [56–58], by regulating proliferation, metastasis, and invasion of cells [59]. However, in MB, the study of the seven IRGs composing the prognostic model is rare, and our research revealed the critical role of these IRGs in MB.

In addition, increasing studies have focused on tumor-infiltrating immune cells and related immunotherapies [17]. A previous study demonstrated that MB patients with high numbers of activated cytotoxic T-lymphocytes (CTLs) have worse survival than patients with low numbers of activated CTLs [25]. Murata’s study proposed that CD8+ tumor-infiltrating lymphocyte is a protective factor for MB [60]. Therefore, we performed CIBERSORT analysis to assess levels of 22 tumor-infiltrating immune cells of MB. Notably, three immune infiltrating cells, including neutrophils, macrophages (M0), and naïve B cells, were significantly associated with the prognostic model risk score, which indicated that the model reflected the MB’s immune status well. Neutrophils are the most abundant group of leukocytes in the blood and essential effectors for inflammation and defense against pathogens [61]. Emerging evidence indicated that neutrophils maintain pro-tumor properties, including enhancement of tumor growth and stimulation of angiogenesis [62]. Recent studies reported that neutrophils could promote immune evasion by suppressing other immune cells, including NK and T cells, the main antitumor cells [63,64]. Additionally, Castriconi’s study proposed MB cell lines are susceptible to lysis by NK cells [23]. Macrophages play essential roles in innate immunity and inflammation [65]. Recently, many studies have demonstrated the protumoral functions of tumor-associated macrophages (TAM). For example, a higher number of TAM is associated with worse clinical prognosis [66]. And a higher TAM level appears to be linked to histological malignancy, cell proliferation, and angiogenesis [67,68]. In our results, neutrophils were found negative to the risk score and activated NK cells, and macrophages were positive to the risk score, consistent with previous studies. Interestingly, among these infiltrating immune cells, naïve B cells were found to be a poor prognostic indicator, negatively associated with risk scores. And memory B cells were the opposite. A recent study demonstrated that B-cell activation and the generation of antibodies are crucial to immunotherapy response, which suggested the critical role of B cells in the progression of tumors [69].

However, there were still some limitations to the present study. First, the lacking of validation *in vitro* and *in vivo* experiments is a limitation of the study. Second, transcriptomics analysis only reflected certain aspects of immune status instead of the overall alterations.

In summary, in the present study, we systematically analyzed the role of IRGs in MB progression and prognosis. Our findings revealed the immune abnormalities of MB. The prognostic model based on IRGs had significant clinical implications for diagnosis and immunotherapy, which could be used in clinical practice.

## Materials and methods

### Microarray data

We screened the data sets containing MB and normal samples in the NCBI-GEO database with a criterion that sample size of MB > 15 and normal tissue > 5. Then, 4 data sets, including 79 MB and 45 normal tissues, were downloaded from the NCBI-GEO database and set as training sets for screening the DE-IRGs. A data set including 605 MB samples with clinical information was set as the training set for survival analysis. Another independent set, including 39 MBs with survival information, was assigned as the test set. The information of these data sets, as shown in Table 2.

**Table 2:** The information of multiple data sets

Data sets	Samples (*n*)		Type	Survival Information	Platform	Reference
	MB*	Normal				
GSE35493	21	9	Training set (DE-IRGs)	No	GPL570	*Birks DK*
GSE50161	22	13		No	GPL570	*Griesinger AM*
GSE66354	19	13		No	GPL570	*Griesinger AM*
GSE86574	17	10		No	GPL570	*Amani V*
GSE85217	605	0	Training set (Sur-IRGs)	Yes	GPL22286	*Cavalli FMG*
GSE12992	39	0	Test set	Yes	GPL570	*Fattet S*

Note: MB for medulloblastoma.

### Screening of DE-IRGs in MB

Each data set was preprocessed separately, including probe definition and normalization. Then differentially expressed genes (DEGs) of four training sets were screened by Limma package in R software comparing MB with normal samples. A false discovery rate (FDR) < 0.05 and a log2 |fold change| > 1 were set as the cutoff values. Then the DEGs were integrated analyzed by the Robust rank aggregation (RRA) algorithm with a score < 0.05. A list of IRGs was derived from the Immunology Database and Analysis Portal (ImmPort) database that updates immunology data accurately and timely. Furthermore, the differentially expressed immune-related genes (DE-IRGs) were selected from DEGs.

### Survival analysis of MB

An independent data set (GSE85217) and clinical information were downloaded and preprocessed by survival package in R software. Then, survival associated immune-related genes (Sur-IRGs) were selected by univariate Cox analysis with *P*-value < 0.05.

### Functional enrichment analysis

To further explore the molecular mechanism in which DE-IRGs and Sur-IRGs were primarily involved. The Gene Ontology (GO) enrichment analysis, including biological process (BP), cellular component (CC), and molecular function (MF), was performed on the DAVID database (https://david.ncifcrf.gov). Immune System Process enrichment analysis was performed by using Cytoscape software. *P*-value < 0.05 was set as cutoff value. Moreover, KEGG pathway analysis was performed and visualized by R software, and results were ranked by *P*-value and count.

### Identification of core IRGs and development of a prognostic signature

Core IRGs were identified by the Venn diagram of DE-IRGs and Sur-IRGs. Then, core IRGs were submitted for multivariate Cox analysis, and a prognostic model was developed based on expression value multiplied by the Cox regression coefficient. Furthermore, the median value of all MBs' risk scores in a dataset was set as the cut-off threshold to define high- and low-risk groups. Additionally, the clinical utility of the prognostic model was evaluated by univariate and multivariate Cox analyses. Receiver operating characteristic (ROC) curves were used to assess the prognostic value of the prognostic model.

### Construction of TF-IRGs regulatory network

In order to clarify the regulatory mechanisms of core IRGs, a list of 318 validated transcription factors (TFs) was derived from the Cistrome Cancer database (http://cistrome.org/CistromeCancer/). And differentially expressed TFs (DE-TFs) in MB were selected from DEGs. Then the relationships between DE-TFs and core IRGs were evaluated by Pearson correlation analysis and visualized by Cytoscape software. The cor cut-off was set to 0.5, and the *P*-value was set to 0.05. The TFs and IRGs in the TF regulatory network were verified in a human transcription factor targets database (hTFtarget, http://bioinfo.life.hust.edu.cn).

### CIBERSORT estimation and correlation analysis

After standard processing, the gene expression data was uploaded to the CIBERSORT web portal (https://cibersort.stanford.edu/index.php), and 1000 permutations were run to assess the content of 22 infiltration immune cells in MB samples. Moreover, Pearson analysis was used to evaluate levels of immune infiltrating cells and the prognostic signature. Besides, univariate Cox analysis was performed to screen the survival-related immune infiltrating cells. The cor cut-off was set to 0.2, and the *P*-value was set to 0.05.

### Statistical analysis

All the data were analyzed and visualized by R software and corresponding packages. Student’s *t*-test was used to compare two groups of data, while one-way ANOVA was used for more than two groups of data. Kaplan–Meier curve and Log-rank test were used for survival analysis.

## Supplementary Material

Supplementary Figure S1-S5 and Table S1

Supplementary Files 1-4

## Data Availability

The data used to support the findings of this study are available from the corresponding author upon request.

## Abbreviations

CAR: chimeric antigen receptor
CSK: C-Terminal Src Kinase
CTL: cytotoxic T-lymphocyte
DEG: differentially expressed gene
DE-TF: differentially expressed TF
EGFR: epidermal growth factor receptor
HDGF: hepatoma-derived growth factor
ICAM2: intercellular adhesion molecule 2
MB: medulloblastoma
ORL1: opioid receptor-like receptor
PNOC: prepronociceptin
ROC: receiver operating characteristic
RORB: retinoic acid-related orphan receptor beta
TAM: tumor-associated macrophages
TF: transcription factor
