# Supplementary material for: Development of a prognostic model based on an immunogenomic landscape analysis of medulloblastoma
Source: Biosci Rep. 2021 Jan 7;41(1):BSR20202907. doi: 10.1042/BSR20202907 (PMC7791544; doi:10.1042/BSR20202907)
Supplement: Supplementary Figure S1-S5 and Table S1 [file BSR-2020-2907_supp.pdf]

Fig. S1

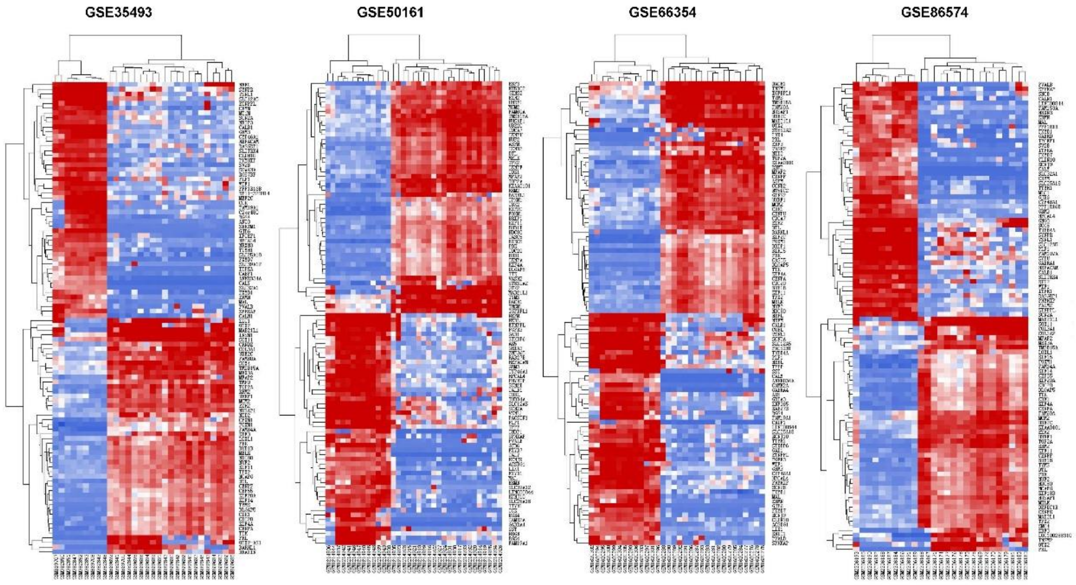

Fig.S1

Heatmaps of top DEGs (25 up- and down-regulated) in each dataset (GSE35493, GSE50161, GSE66354 and GSE86574).

Fig. S2

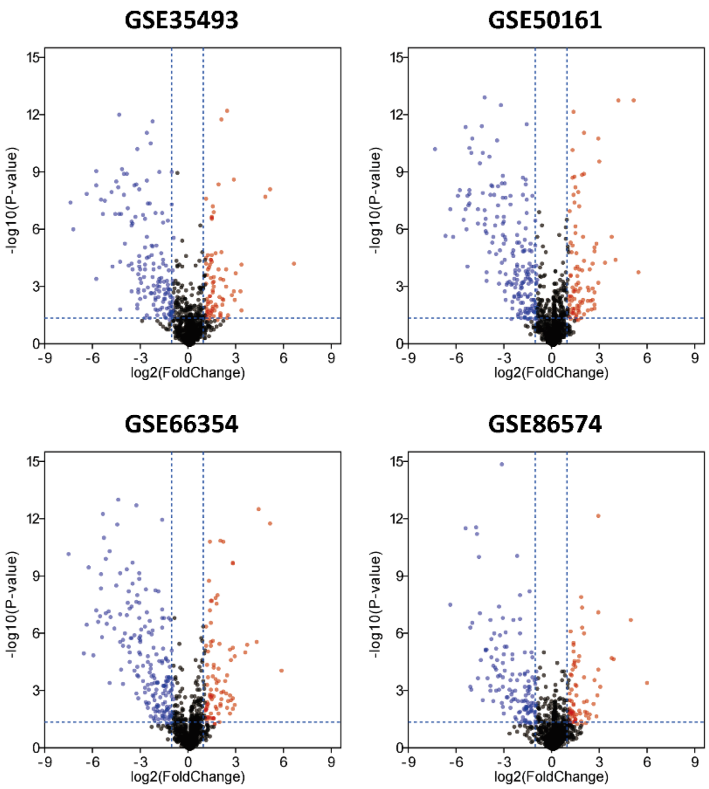

Fig.S2

Volcanoplots of differentially expressed IRGs (Red for Up-regulated and blue for down-regulated).

Fig. S3

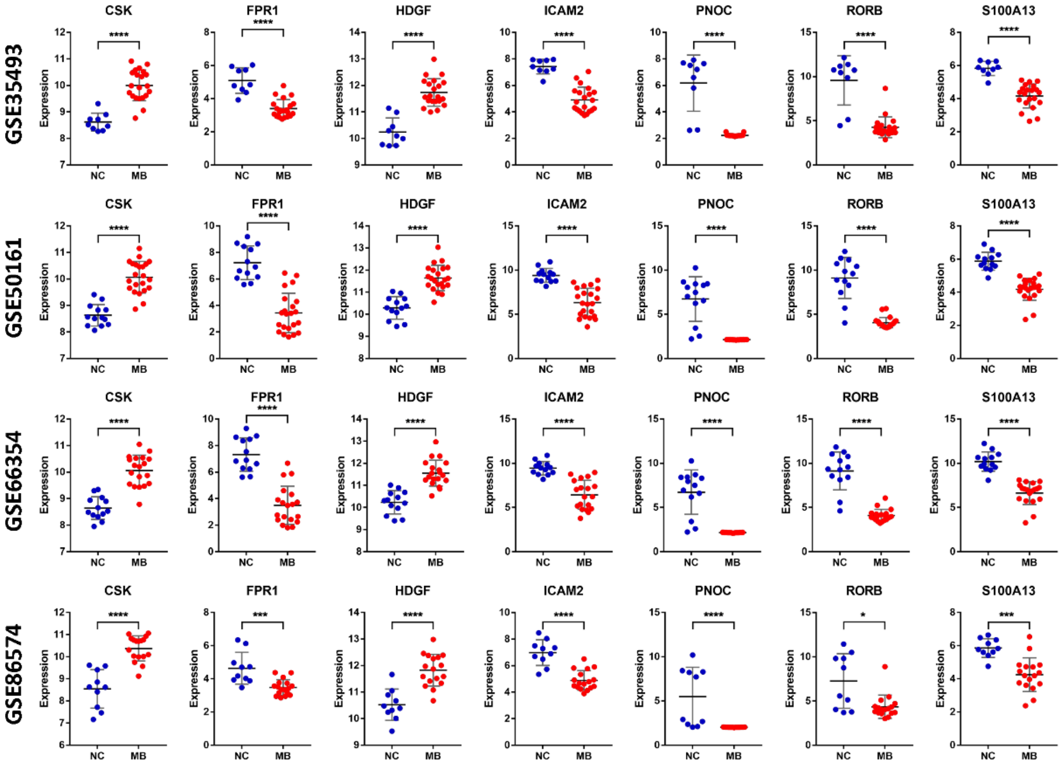

Fig.S3

Expression of seven core genes in the four training datasets.

Fig.S4

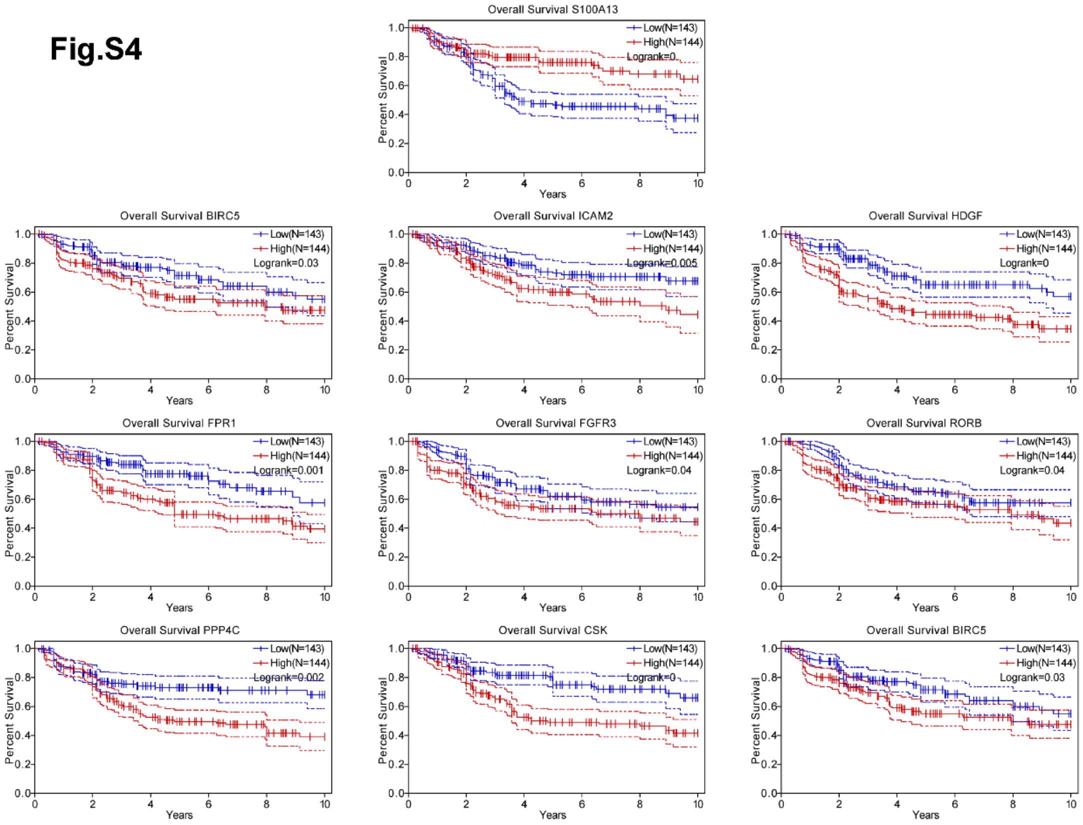

Fig.S4

Survival plots of ten core genes.

Fig. S5

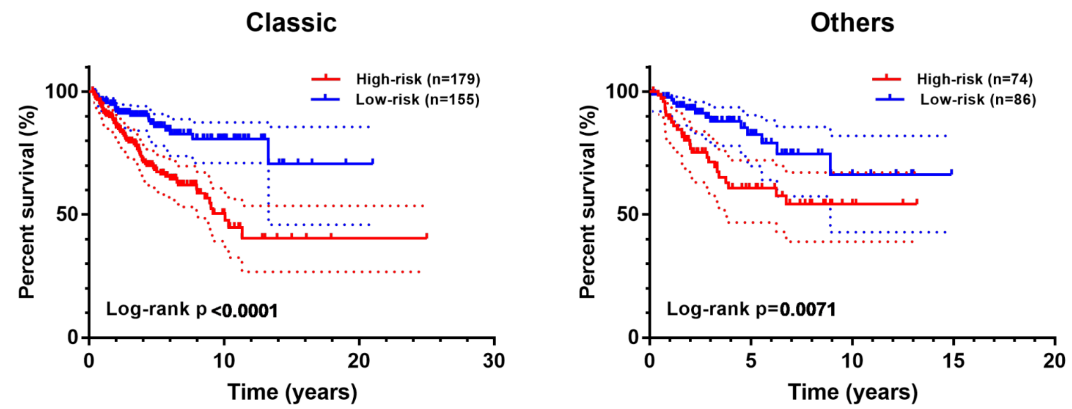

Fig.S5

Survival analysis of the model in Classic MBs and the others.

Table. S1

The information of the core genes.

Gene	Molecular Function	Subcellular Location
**HDGF**	Acts as a transcriptional repressor and has mitogenic activity for fibroblasts.	Nucleus
**CSK**	Non-receptor tyrosine-protein kinase that plays an important role in the regulation of cell growth, differentiation, migration, and immune response.	Plasma membrane
**PNOC**	Ligand of the opioid receptor-like receptor OPRL1. It may act as a transmitter in the brain by modulating nociceptive and locomotor behavior. May be involved in neuronal differentiation and development.	Extracellular
**S100A13**	It plays a role in the export of proteins that lack a signal peptide and are secreted by an alternative pathway.	Plasma membrane, Extracellular, Nucleus, Cytosol
**RORB**	The nuclear receptor binds DNA as a monomer to ROR response elements (RORE) containing a single core motif half-site 5'-AGGTCA-3' preceded by a short A-T-rich sequence.	Nucleus
**FPR1**	High affinity receptor for N-formyl-methionyl peptides (fMLP), which are powerful neutrophil chemotactic factors.	Lysosome
**ICAM2**	Ligands for the leukocyte adhesion protein LFA-1 (integrin alpha-L/beta-2). ICAM2 may play a role in lymphocyte recirculation by blocking LFA-1-dependent cell adhesion.	Plasma membrane
